# Evidence for Transfer of Membranes from Mesenchymal Stem Cells to HL-1 Cardiac Cells

**DOI:** 10.1155/2014/653734

**Published:** 2014-09-09

**Authors:** Robert A. Boomsma, David L. Geenen

**Affiliations:** ^1^Department of Biology, Trinity Christian College, Palos Heights, IL 60463, USA; ^2^Physician Assistant Studies/Allied Health Sciences, College of Health Professions, Grand Valley State University, Grand Rapids, IL 49503, USA

## Abstract

This study examined the interaction of mouse bone marrow mesenchymal stem cells (MSC) with cardiac HL-1 cells during coculture by fluorescent dye labeling and then flow cytometry. MSC were layered onto confluent HL-1 cell cultures in a 1 : 4 ratio. MSC gained gap junction permeant calcein from HL-1 cells after 4 hours which was partially reduced by oleamide. After 20 hours, 99% MSC gained calcein, unaffected by oleamide. Double-labeling HL-1 cells with calcein and the membrane dye DiO resulted in transfer of both calcein and DiO to MSC. When HL-1 cells were labeled with calcein and MSC with DiO, MSC gained calcein while HL-1 cells gained DiO. Very little fusion was observed since more than 90% Sca-1 positive MSC gained DiO from HL-1 cells while less than 9% gained gap junction impermeant CMFDA after 20 hours with no Sca-1 transfer to HL-1 cells. Time dependent transfer of membrane DiD was observed from HL-1 cells to MSC (100%) and vice versa (50%) after 20 hours with more limited transfer of CMFDA. These results demonstrate that MSC and HL-1 cells exchange membrane components which may account for some of the beneficial effect of MSC in the heart after myocardial infarction.

## 1. Introduction

We have previously shown that injection of mouse bone marrow mesenchymal stem cells (MSC) prevents the loss of function that occurs in mouse hearts after coronary artery occlusion [1]. The mechanism for this protective effect is unclear since there was no reduction in ventricular scarring or evidence of cardiomyocyte differentiation in integrated MSC. We have recently shown that MSC secrete a variety of cytokines that have a significant effect on angiogenesis, apoptosis, and cell migration [2] supporting the hypothesis that MSC protect the heart, at least in part, by secreting paracrine factors [3].

Ventricular myocytes exhibit extensive gap junctions formation [4] which provides electrical coupling and the transfer of small molecules between cells (<1 kD) [5, 6]. MSC express connexins and are able to form gap junctions with cardiac ventricular myocytes [7] and HL-1 cardiac cells [8]. Thus, gap junctions may provide a conduit for the cardioprotective effects of MSC in the heart [8, 9].

In addition, cells are able to communicate with the exchange of cytoplasmic and membrane components by the formation of tunneling nanotubes [10] or extracellular vesicles [11]. Nanotubes are 50–200 nm diameter membranous channels containing F-actin that form a cytoplasmic connection between cells [12]. Extracellular vesicles are heterogeneous and include exosomes, microvesicles, and ectosomes which vary by their size and cellular origin [13, 14]. In addition to the secretion of cytokines, nanotubes and vesicles may play an important role in the cardioprotective effect of MSC in the heart [15–19].

In the current study, we examined the interaction of MSC with cardiac HL-1 cells in a coculture system. Although we were looking for the transfer of small cytoplasmic components via gap junctions, we found significant transfer of membrane components.

## 2. Methods

### 2.1. MSC and HL-1 Cell Culture

Mouse bone marrow mesenchymal stem cells (MSC; passages 20–25) were cultured in 10 cm plates at 1.0 × 10^6^ cells/plate as previously described [1] in complete mouse Mesencult media (basal media + stimulatory supplement; Stem Cell Technologies) until confluent. Cells were then fluorescently labeled and subsequently lifted with 0.25% trypsin-EDTA for coculture with HL-1 cells.

Cardiac HL-1 cells were generously supplied by W.C. Claycomb and cultured according to previously published specifications [20]. Cells were cultured in 6-well plates coated with 0.02% gelatin + 0.05% fibronectin at 0.8 × 10^6^ cells/well in complete Claycomb media (Claycomb media (Sigma) + 10% FBS + 0.1 mM norepinephrine + 2 mM glutamine + 100 U/mL penicillin + 100 *μ*g/mL streptomycin) until the cells were confluent. Cells were then fluorescently labeled prior to coculture with MSC.

MSC or HL-1 monolayers were labeled with various fluorescent dyes prior to coculture. Cells were washed 2 times in PBS to remove serum and then incubated with dye in DMEM (MSC) or Claycomb base media (HL-1) containing no serum or other additives. Following dye labeling, cells were washed in PBS. Cells were subsequently labeled with 2.5 *μ*M of the cytoplasmic gap junction permeant calcein red-orange AM (Cell Trace; Molecular Probes/Invitrogen, C34851) for 1 hour, 5 *μ*M of the cytoplasmic gap junction impermeant chloromethyl fluorescein diacetate (Cell Tracker Green CMFDA; Molecular Probes/Invitrogen, C7025) for 1 hour (with a media replacement after 30 minutes), or 5 *μ*M of the membrane labeling carbocyanine dyes DiO or DiD (Vibrant Cell Labeling Solution; Molecular Probes/Invitrogen, V-22886, V-22887) for 1 hour. In addition, in some experiments MSC were labeled in suspension with anti-mouse-Sca1-PEcy5 (eBiosciences, 15-5981) for 1 hr and washed in PBS + 0.25% BSA+ 2 mM EDTA prior to use. Controls were incubated with a mouse isotype control instead.

After labeling, MSC and HL-1 cells were cocultured for 4 or 20 hours. MSC were resuspended in complete Claycomb media and 0.25 × 10^6^ MSC were layered onto confluent HL-1 cultures (1.0–1.5 × 10^6^ HL-1 cells/well) in 6-well plates. Following coculture, cells were washed in PBS, lifted with 0.25% trypsin-EDTA, centrifuged, resuspended in 3 mL PBS/BSA/EDTA, passed through a 100 *μ*M filter, centrifuged, and resuspended in 100 *μ*L PBS/BSA/EDTA for analysis by flow cytometry. Flow cytometry was performed in the UIC Research Resources Center, Flow Cytometry Service, using the BD LSRFortessa Cell Analyzer. Data was analyzed using Summit 4.3 software. All coculture experiments were replicated at least 4 times and the percentage of labeled cells was determined as mean ± s.e.m.; significance was assessed using Student's *t*-test.

### 2.2. H9c2 Cell Scrape Loading

Cardiac myoblast H9c2 cells (ATCC number CRL-1446) were cultured in fibronectin/gelatin coated 6-well plates at 0.3 × 10^6^ cells/well in DMEM + 10% FBS for 72 hours in the presence or absence of 50 *μ*M oleamide. Confluent cultures were then scrape loaded with Lucifer yellow or dextran-Rhodamine B [6]. Specifically, the wells were washed twice with calcium-free PBS and then 0.05% Lucifer yellow dipotassium salt (Sigma) or 0.1% dextran-Rhodamine B (10,000 MW; Invitrogen) in calcium-free PBS was added. After scraping a single line with a 29 gauge needle and incubating for 7 minutes, the cultures were washed three times in PBS + 1.5 mM Ca^++^ and observed using an inverted fluorescent microscope.

## 3. Results

### 3.1. Detection of Gap Junctions

**Figure 1:**
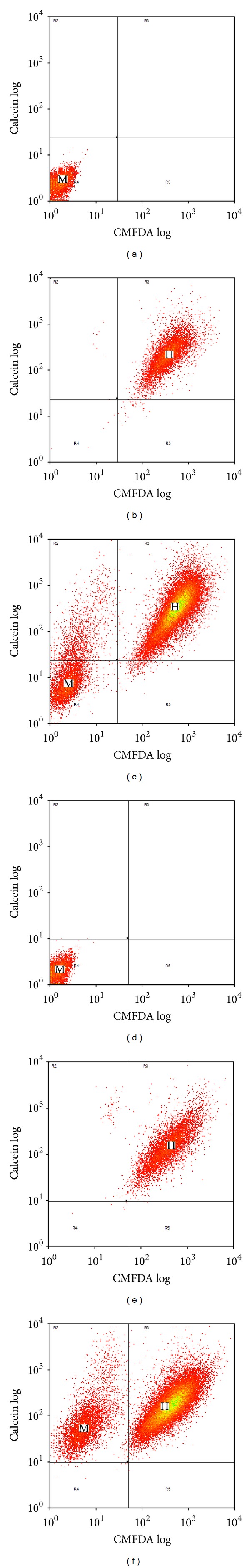
Flow cytometry of unlabeled MSC and CMFDA/calcein labeled HL-1 cells after 4 and 20 hours of coculture. (a–c) Four hours of coculture. (d–f) 20 hours of coculture. (a) and (d) Unlabeled MSC (M) prior to coculture. (b) and (e) HL-1 cells (H) double labeled with CMFDA and calcein prior to coculture. (c) and (f) Unlabeled MSC and CMFDA/calcein labeled HL-1 cells after coculture.

In order to study the formation of gap junctions between MSC and HL-1 cells, confluent cultures of HL-1 cells were double labeled with the cytoplasmic gap junction impermeant dye CMFDA and the gap junction permeant dye calcein red-orange AM (Figure 1). Unlabeled MSC were then layered on top of the confluent labeled HL-1 cells in a 1 : 4 ratio to mimic the lower number of transplanted MSC compared to endogenous myocytes when used* in vivo*. Analysis by flow cytometry after 4 hours of coculture demonstrated that 23.3 ± 2.2% of the MSC gained calcein while no CMFDA was transferred (Figures 1(a)–1(c)). Treatment of MSC with 50 *μ*M oleamide, a gap junction blocker [21, 22], for 10 minutes prior to and during coculture significantly reduced the amount of calcein transfer to 16.6 ± 2.0%, indicating that at least some transfer of calcein was due to the formation of gap junctions. Coculture for 20 hours resulted in transfer of calcein to 99.8 ± 0.1% of MSC (Figures 1(d)–1(f)); interestingly, oleamide treatment had no effect after 20 hours, suggesting that gap junctions were not involved in calcein transfer after 20 hours.

**Figure 2:**
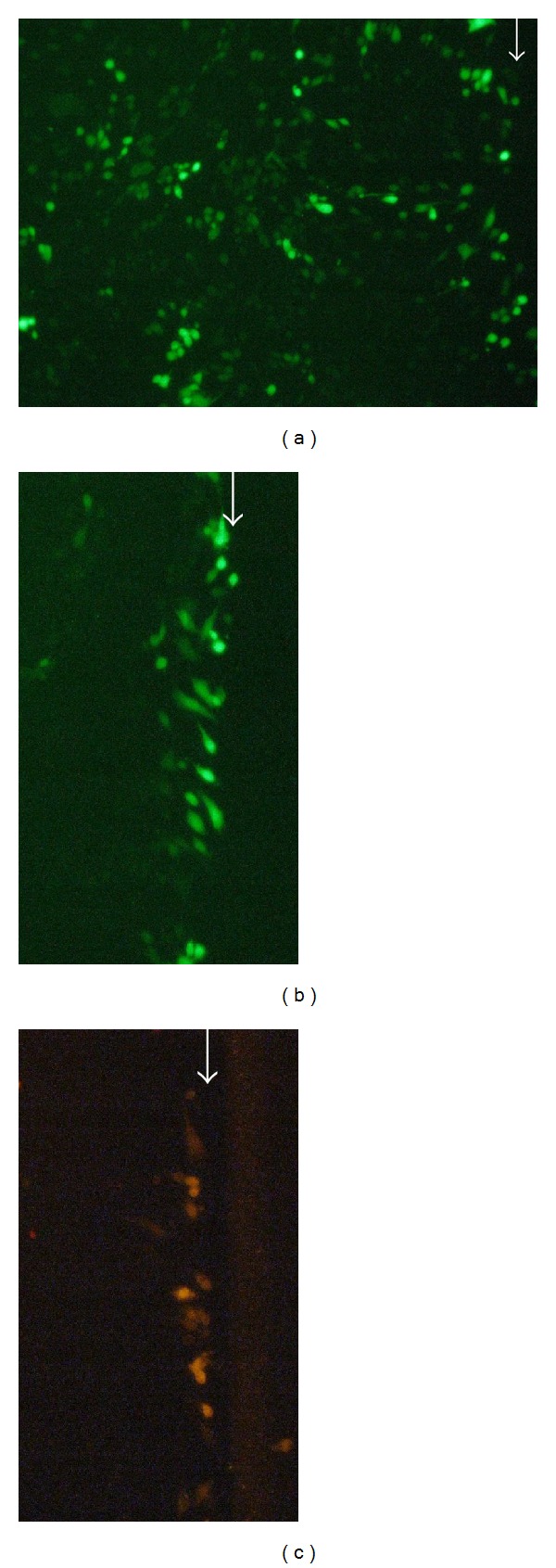
Scrape loading of H9c2 cells. (a) Scrape loading with Lucifer yellow in the absence of oleamide. (b) Scrape loading with Lucifer yellow in the presence of oleamide. (c) Scrape loading with dextran-Rhodamine B in the absence of oleamide. Arrow: edge of scrape.

Since oleamide did not completely block calcein transfer, we wanted to confirm that 50 *μ*M oleamide is sufficient to block gap junctions. Cardiac H9c2 cells, known to form functional gap junctions [23], were cultured in the presence or absence of 50 *μ*M oleamide and then scrape loaded with Lucifer yellow upon confluence. In the absence of oleamide, Lucifer yellow was transferred 10–30 cell layers away from the scrape (Figure 2(a)). In the presence of oleamide, Lucifer yellow was only transferred 1-2 cell layers from the scrape (Figure 2(b)). Scrape loading with gap junction impermeant dextran-Rhodamine B demonstrated a lack of dye transfer to any cell layers away from the scrape (Figure 2(c)). These results demonstrate that 50 *μ*M oleamide is sufficient to block gap junctions.

**Figure 3:**
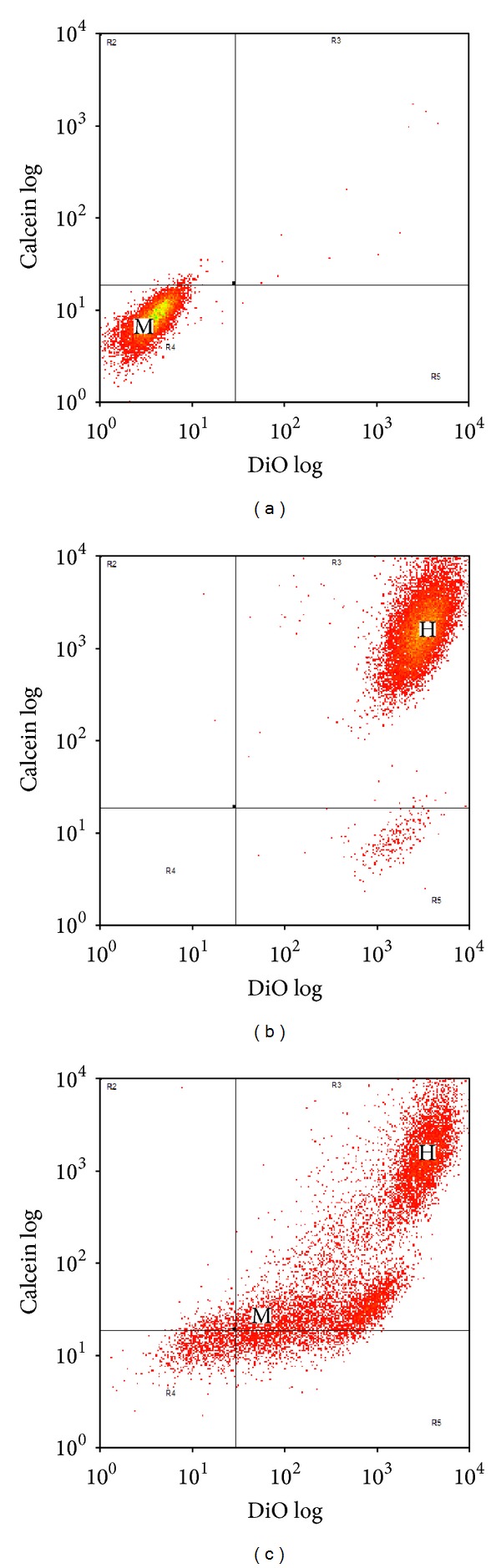
Flow cytometry of unlabeled MSC and DiO/calcein labeled HL-1 cells after 4 hours of coculture. (a) Unlabeled MSC (M) prior to coculture. (b) HL-1 cells (H) double labeled with DiO and calcein prior to coculture. (c) Unlabeled MSC and DiO/calcein labeled HL-1 cells after 4 hours of coculture.

In separate experiments, confluent cultures of HL-1 cells were double labeled with the membrane dye DiO along with calcein and then unlabeled MSC were layered on top in a 1 : 4 ratio as before and cocultured for 4 hours. After flow cytometry, a completely different pattern was observed (Figure 3). Not only was there a shift in fluorescence of the MSC population denoting a gain of calcein as before but also the MSC population appeared to gain DiO suggesting a transfer of membrane.

**Figure 4:**
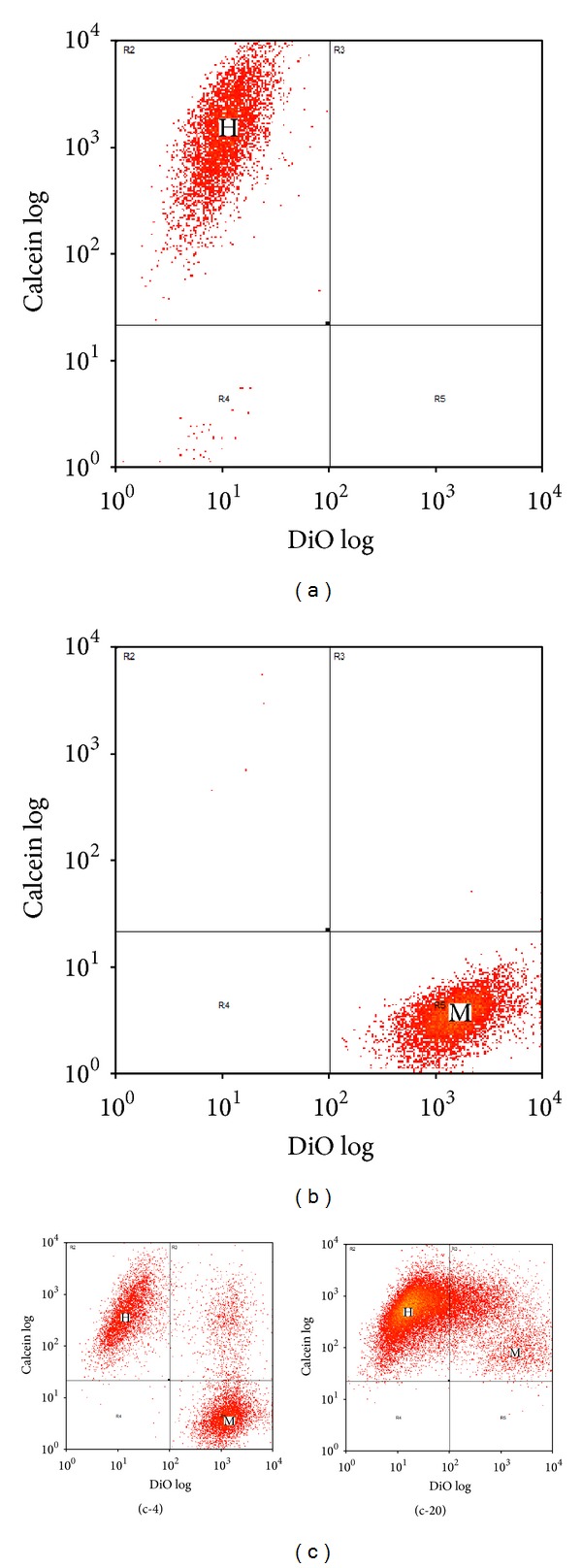
Flow cytometry of DiO labeled MSC and calcein labeled HL-1 cells after 4 and 20 hours of coculture. (a) HL-1 cells (H) labeled with calcein prior to coculture. (b) MSC (M) labeled with DiO prior to coculture. (c-4) DiO labeled MSC and calcein labeled HL-1 cells after 4 hours of coculture. (c-20) DiO labeled MSC and calcein labeled HL-1 cells after 20 hours of coculture.

In order to further assess the phenomenon of a gain in DiO, confluent cultures of HL-1 cells were labeled with calcein while MSC were labeled with DiO. The MSC were then layered onto the HL-1 cells in a 1 : 4 ratio and cocultured for 4 and 20 hours (Figure 4). After 4 hours some of the MSC gained calcein from the HL-1 cells (Figure 4(c-4)). However, after 20 hours (Figure 4(c-20)), all of the MSC gained cytoplasmic calcein from the HL-1 cells, while some of the HL-1 cells also gained membrane DiO from the MSC.

We concluded from these experiments that gap junctions could account for the transfer of some calcein. However, the data suggests that another mechanism was also responsible since oleamide was unable to completely block calcein transfer after 4 hours and was ineffective after 20 hours. In addition, membrane components (DiO) were being transferred after 4 and 20 hours.

### 3.2. Test for Fusion of MSC with HL-1 Cells

**Figure 5:**
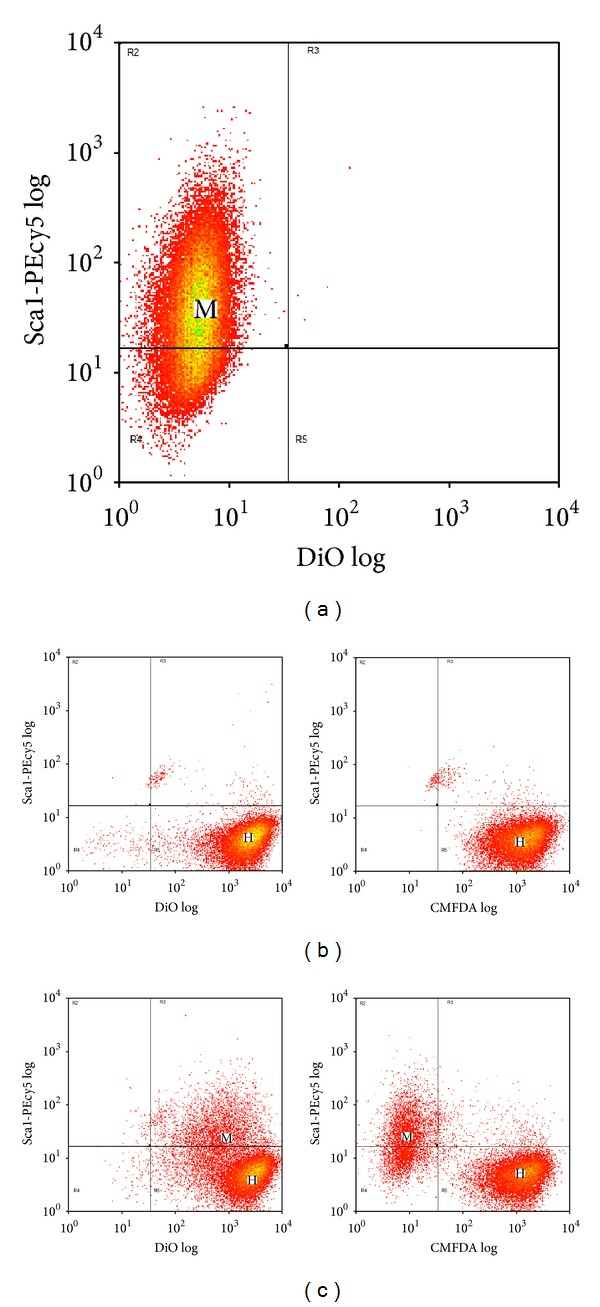
Flow cytometry of Sca1-PEcy5 labeled MSC and DiO or CMFDA labeled HL-1 cells after 20 hours of coculture. (a) MSC (M) labeled with Sca1-PEcy5 prior to coculture. (b) DiO: HL-1 cells (H) labeled with DiO prior to coculture. (c) DiO: Sca1-PEcy5 labeled MSC and DiO labeled HL-1 cells after 20 hours of coculture. (b) CMFDA: HL-1 cells (H) labeled with CMFDA prior to coculture. (c) CMFDA: Sca1-PEcy5 labeled MSC and CMFDA labeled HL-1 cells after 20 hours of coculture.

We reasoned that MSC might be fusing with HL-1 cells, causing the transfer of membrane components between MSC and HL-1 cells. In order to test this hypothesis, MSC were labeled with the stem cell membrane marker Sca1 using anti-Sca1-PEcy5. HL-1 cells were labeled with either membrane DiO or cytoplasmic CMFDA. MSC were then layered onto confluent HL-1 cultures in a 1 : 4 ratio, cocultured for 20 hours, and analyzed by flow cytometry (Figure 5). We theorized that if fusion occurred, then both DiO and CMFDA would be transferred to the MSC labeled with Sca1. However, if fusion did not occur, then the cytoplasmic CMFDA would remain limited to the HL-1 cells. As shown in Figure 5(a), 76% of the MSC were Sca1 positive. Coculture with DiO labeled HL-1 cells demonstrated 92.4 ± 0.6% transfer of membrane DiO to Sca1 positive MSC (Figure 5(b), DiO; Figure 5(c), DiO). Coculture with CMFDA labeled HL-1 cells showed only 8.8 ± 0.5% of the Sca1 positive MSC gained cytoplasmic CMFDA (Figure 5(b), CMFDA; Figure 5(c), CMFDA). There was no apparent transfer of Sca1 to HL-1 cells.

These results indicated to us that there may be a small amount of fusion of MSC with HL-1 cells due to the limited transfer of cytoplasm; however, there was a large transfer of membrane components from HL-1 cells to MSC.

### 3.3. Test for Membrane Transfer between MSC and HL-1 Cells

**Figure 6:**
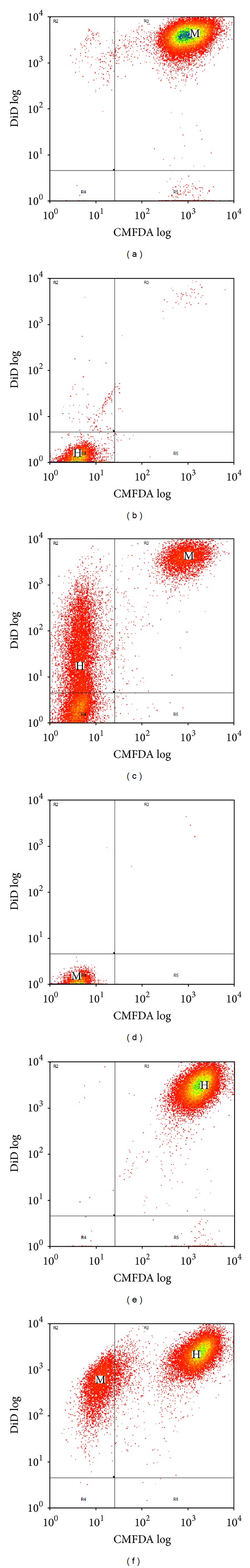
Flow cytometry of DiD/CMFDA labeled MSC and unlabeled HL-1 cells or unlabeled MSC and DiD/CMFDA labeled HL-1 cells after 20 hours of coculture. (a) MSC (M) labeled with DiD and CMFDA prior to coculture. (b) Unlabeled HL-1 cells (H) prior to coculture. (c) DiD/CMFDA labeled MSC and unlabeled HL-1 cells after 20 hours of coculture. (d) Unlabeled MSC (M) prior to coculture. (e) HL-1 cells (H) labeled with DiD and CMFDA prior to coculture. (f) Unlabeled MSC and DiD/CMFDA HL-1 cells after 20 hours of coculture.

To determine the time course and amount of membrane transfer, MSC were layered onto confluent cultures of HL-1 cells in a 1 : 4 ratio after labeling with different combinations of membrane DiD and cytoplasmic CMFDA. In one group the MSC were double labeled with membrane DiD and cytoplasmic CMFDA and the HL-1 cells were unlabeled. In the second group, the HL-1 cells were double labeled while the MSC were unlabeled (Figure 6). Since our goal was to monitor membrane transfer, CMFDA was used to label the cytoplasm because cytoplasmic calcein was readily transferred between cells. The cells were then cocultured for 4 and 20 hours.

**Figure 7:**
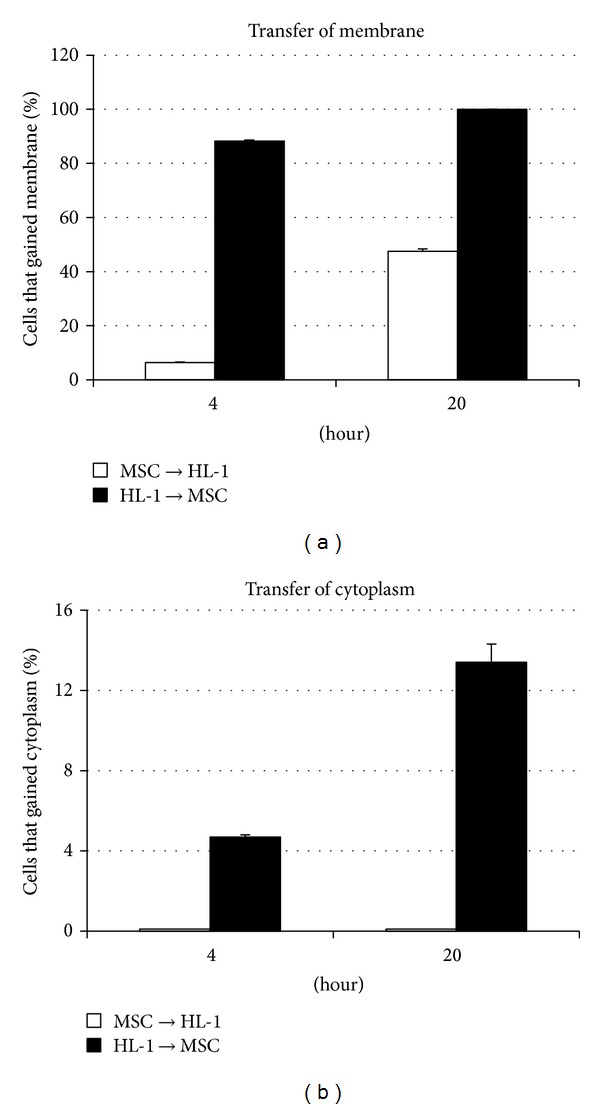
Percent cells that gained membrane or cytoplasm after 4 and 20 hours of coculture of MSC and HL-1 cells. MSC and HL-1 cells were labeled, cultured, and analyzed as in Figure 6. Data was calculated as mean percent ± s.e.m. of cells that gained membrane (DiD label) or cytoplasm (CMFDA label) during coculture as determined by flow cytometry. MSC→HL-1 = transfer from MSC to HL-1 cells (open bars). HL-1→MSC = transfer from HL-1 cells to MSC (solid bars).

When the MSC were double labeled, coculture with unlabeled HL-1 cells for 20 hours (Figures 6(a)–6(c), Figure 7) demonstrated that 47.5 ± 0.8% of the HL-1 cells gained membrane DiD from the MSC; there was no detectable transfer of cytoplasmic CMFDA from the MSC to the HL-1 cells. After 4 hours (Figure 7) only 6.4 ± 0.2% unlabeled HL-1 gained membrane DiD from double labeled MSC; again, these cells did not pick up any cytoplasmic CMFDA.

When the MSC were unlabeled and HL-1 cells were double labeled, coculture for 20 hours (Figures 6(d)–6(f), Figure 7) demonstrated that 100% of the MSC gained membrane DiD from the HL-1 cells. In addition, 13.4 ± 0.9% of the MSC gained cytoplasmic CMFDA from the HL-1 cells. After 4 hours (Figure 7), 88.2 ± 0.4% unlabeled MSC gained membrane DiD from double labeled HL-1 cells; 4.7 ± 0.1% of the MSC gained cytoplasmic CMFDA.

## 4. Discussion

This study clearly demonstrates for the first time that MSC and HL-1 cells exchange membrane components in a time-dependent manner when cocultured. We found that 100% of the MSC gained membrane from the HL-1 cells after 20 hours while 48% of the HL-1 cells gained membrane from the MSC. This smaller amount of membrane component transfer to HL-1 cells is consistent with our experimental design since there were 80% fewer MSC than HL-1 cells resulting in a lower probability for MSC to exchange membrane with HL-1 cells. It is interesting that while the membrane lipid dyes DiO and DiD transferred between the cells, the Sca1 membrane protein did not transfer from the MSC to the HL-1 cells. One plausible explanation for this finding is a selective transfer of membrane components [13] between MSC and HL-1 cells. It is probable that the membranes were exchanged due to the close contact between the MSC and HL-1 cells during coculture since MSC were layered on top of confluent HL-1 cells. Possible cellular mechanisms that could account for this exchange include tunneling nanotubes as seen between MSC and cardiomyocytes [24] or the exchange of exosomes or microvesicles, consistent with a previous report of MSC and nucleus pulposis cell coculture [25].

This study also suggests the formation of gap junctions between MSC and HL-1 cells during the early stages of coculture. After 4 hours some of the calcein dye transfer was oleamide sensitive. This is consistent with the work of Mureli and coworkers [8] who found that MSC began to form gap junctions within 20 minutes when cocultured with HL-1 cells, 46% formed junctions after 4 hours and 60% after 24 hours. In our study 100% of the MSC were found to have gained calcein from the HL-1 cells after 20 hours, a transfer that was oleamide insensitive. This suggests that calcein transfer was initially due, in part, to gap junctions but later occurred by alternate means most likely in conjunction with the observed membrane transfer.

We found that approximately 10% of MSC gained the gap junction impermeant dye CMFDA from HL-1 cells indicating a low level of fusion between MSC and HL-1 cells. Although we did not detect CMFDA transfer from MSC to HL-1 cells, this is most likely due to the lower numbers of MSC compared to HL-1 cells. Whether or not fusion of MSC with resident cells accounts for observed transdifferentiation of MSC into cardiomyocytes has been a persistent question [26, 27]. Our results, coupled with those for cardiomyocytes [28], neural cells [29], and the nucleus pulposis [25] show that fusion events, if they occur at all, are quite rare.

It now appears that the cardioprotective effect of MSC may be multifaceted. Although there is evidence that MSC are able to differentiate into a cardiomyocyte phenotype [30–32], much of the beneficial effect is probably due to alternate mechanisms [33]. First, work in our laboratory [2] and others [3, 15] has clearly shown that MSC secrete a variety of cytokines that are able to affect migration, angiogenesis, apoptosis, and the immune reaction. Second, the current study along with that of Mureli et al. [8] demonstrated the rapid formation of gap junctions after coculture. Gap junctions not only provide electrical coupling between MSC and cardiomyocytes [7, 8] but also could function to exchange other molecules that are important in the differentiation of MSC into a cardiomyocyte lineage [34]. Third, paracrine communication by MSC via extracellular vesicles (exosomes and microvesicles) is now well established [11], and exosomes from MSC have a beneficial effect on the heart after ischemia-reperfusion injury [16]. These vesicles are able to transport a wide variety of molecules such as membrane components, mRNA, miRNA, and signaling molecules between various types of cells [13, 14]. This exosomal transfer may be bidirectional since cardiomyocytes produce exosomes containing a wide variety of mRNA that affect fibroblast gene transcription [35]. Fourth, it is becoming increasingly clear that cells are able to communicate through the formation of tunneling nanotubes [12]. Nanotubes allow for electrical coupling and the transfer of membrane and cytoplasmic components between cells [10, 36]. Nanotube connections between MSC and cardiomyocytes [24] allow the transfer of mitochondria [17], revert the differentiation state of cardiomyocytes toward a progenitor state [18], and alter the secretion of paracrine factors by MSC [19]. The membrane transfer observed in our study may be due to the exchange of extracellular vesicles or the formation of tunneling nanotubes between MSC and HL-1 cells.

## 5. Conclusion

This study demonstrates that MSC are able to exchange membrane components with HL-1 cardiac cells. Exchange of this nature may account for some of the beneficial effect we observed when MSC were injected into mice with surgically induced permanent coronary artery occlusion [1] in addition to the previously reported secretion of cytokines by MSC [2]. Future studies will focus on the mechanism of transfer and the specific molecules exchanged between these cells and their potential function.

## References

[1] Boomsma RA, Swaminathan PD, Geenen DL (2007). Intravenously injected mesenchymal stem cells home to viable myocardium after coronary occlusion and preserve systolic function without altering infarct size. *International Journal of Cardiology*.

[2] Boomsma RA, Geenen DL (2012). Mesenchymal stem cells secrete multiple cytokines that promote angiogenesis and have contrasting effects on chemotaxis and apoptosis. *PloS one*.

[3] Burchfield JS, Dimmeler S (2008). Role of paracrine factors in stem and progenitor cell mediated cardiac repair and tissue fibrosis. *Fibrogenesis Tissue Repair*.

[4] Saffitz JE, Hames KY, Kanno S (2007). Remodeling of gap junctions in ischemic and nonischemic forms of heart disease. *Journal of Membrane Biology*.

[5] Kar R, Batra N, Riquelme MA, Jiang JX (2012). Biological role of connexin intercellular channels and hemichannels. *Archives of Biochemistry and Biophysics*.

[6] Abbaci M, Barberi-Heyob M, Blondel W, Guillemin F, Didelon J (2008). Advantages and limitations of commonly used methods to assay the molecular permeability of gap junctional intercellular communication. *BioTechniques*.

[7] Valiunas V, Doronin S, Valiuniene L (2004). Human mesenchymal stem cells make cardiac connexins and form functional gap junctions. *The Journal of Physiology*.

[8] Mureli S, Gans CP, Bare DJ, Geenen DL, Kumar NM, Banach K (2013). Mesenchymal stem cells improve cardiac conduction by upregulation of connexin 43 through paracrine signaling. *The American Journal of Physiology: Heart and Circulatory Physiology*.

[9] Hahn J-Y, Cho H-J, Kang H-J (2008). Pre-treatment of mesenchymal stem cells with a combination of growth factors enhances gap junction formation, cytoprotective effect on cardiomyocytes, and therapeutic efficacy for myocardial infarction. *Journal of the American College of Cardiology*.

[10] Abounit S, Zurzolo C (2012). Wiring through tunneling nanotubes—from electrical signals to organelle transfer. *Journal of Cell Science*.

[11] Katsuda T, Kosaka N, Takeshita F, Ochiya T (2013). The therapeutic potential of mesenchymal stem cell-derived extracellular vesicles. *Proteomics*.

[12] Gerdes H-H, Rustom A, Wang X (2013). Tunneling nanotubes, an emerging intercellular communication route in development. *Mechanisms of Development*.

[13] Mause SF, Weber C (2010). Microparticles: protagonists of a novel communication network for intercellular information exchange. *Circulation Research*.

[14] Mittelbrunn M, Sánchez-Madrid F (2012). Intercellular communication: diverse structures for exchange of genetic information. *Nature Reviews Molecular Cell Biology*.

[15] Liang X, Ding Y, Zhang Y, Tse HF, Lian Q (2013). Paracrine mechanisms of Mesenchymal Stem cell-based therapy: current status and perspectives. *Cell Transplant*.

[16] Arslan F, Lai RC, Smeets MB (2013). Mesenchymal stem cell-derived exosomes increase ATP levels, decrease oxidative stress and activate PI3K/Akt pathway to enhance myocardial viability and prevent adverse remodeling after myocardial ischemia/reperfusion injury. *Stem Cell Research*.

[17] Plotnikov EY, Khryapenkova TG, Vasileva AK (2008). Cell-to-cell cross-talk between mesenchymal stem cells and cardiomyocytes in co-culture. *Journal of Cellular and Molecular Medicine*.

[18] Acquistapace A, Bru T, Lesault P-F (2011). Human mesenchymal stem cells reprogram adult cardiomyocytes toward a progenitor-like state through partial cell fusion and mitochondria transfer. *Stem Cells*.

[19] Figeac F, Lesault PF, Le Coz O (2014). Nanotubular crosstalk with distressed cardiomyocytes stimulates the paracrine repair function of mesenchymal stem cells. *Stem Cells*.

[20] Claycomb WC, Lanson NA, Stallworth BS (1998). HL-1 cells: A cardiac muscle cell line that contracts and retains phenotypic characteristics of the adult cardiomyocyte. *Proceedings of the National Academy of Sciences of the United States of America*.

[21] Boger DL, Patterson JE, Guan X, Cravatt BF, Lerner RA, Gilula NB (1998). Chemical requirements for inhibition of gap junction communication by the biologically active lipid oleamide. *Proceedings of the National Academy of Sciences of the United States of America*.

[22] Juszczak GR, Swiergiel AH (2009). Properties of gap junction blockers and their behavioural, cognitive and electrophysiological effects: animal and human studies. *Progress in Neuro-Psychopharmacology & Biological Psychiatry*.

[23] Zou J, Yue XY, Zheng SC (2014). Cholesterol modulates function of connexin 43 gap junction channel via PKC pathway in H9c2 cells. *Biochimica et Biophysica Acta*.

[24] Ma Z, Yang H, Liu H (2013). Mesenchymal stem cell-cardiomyocyte interactions under defined contact modes on laser-patterned biochips. *PLoS ONE*.

[25] Strassburg S, Hodson NW, Hill PI, Richardson SM, Hoyland JA (2012). Bi-directional exchange of membrane components occurs during co-culture of mesenchymal stem cells and nucleus pulposus cells. *PLoS ONE*.

[26] Dimmeler S, Zeiher AM, Schneider MD (2005). Unchain my heart: the scientific foundations of cardiac repair. *Journal of Clinical Investigation*.

[27] Alvarez-Dolado M, Pardal R, Garcia-Verdugo JM (2003). Fusion of bone-marrow-derived cells with Purkinje neurons, cardiomyocytes and hepatocytes. *Nature*.

[28] Tsuji H, Miyoshi S, Ikegami Y (2010). Xenografted human amniotic membrane-derived mesenchymal stem cells are immunologically tolerated and transdifferentiated into cardiomyocytes. *Circulation Research*.

[29] Maltman DJ, Hardy SA, Przyborski SA (2011). Role of mesenchymal stem cells in neurogenesis and nervous system repair. *Neurochemistry International*.

[30] Grajales L, García J, Banach K, Geenen DL (2010). Delayed enrichment of mesenchymal cells promotes cardiac lineage and calcium transient development. *Journal of Molecular and Cellular Cardiology*.

[31] Grajales L, García J, Geenen DL (2012). Induction of cardiac myogenic lineage development differs between mesenchymal and satellite cells and is accelerated by bone morphogenetic protein-4. *Journal of Molecular and Cellular Cardiology*.

[32] Choi Y-H, Kurtz A, Stamm C (2011). Mesenchymal stem cells for cardiac cell therapy. *Human Gene Therapy*.

[33] Gnecchi M, Zhang Z, Ni A, Dzau VJ (2008). Paracrine mechanisms in adult stem cell signaling and therapy. *Circulation Research*.

[34] Ramkisoensing AA, Pijnappels DA, Swildens J (2012). Gap junctional coupling with cardiomyocytes is necessary but not sufficient for cardiomyogenic differentiation of cocultured human mesenchymal stem cells. *Stem Cells*.

[35] Waldenström A, Gennebäck N, Hellman U, Ronquist G (2012). Cardiomyocyte microvesicles contain DNA/RNA and convey biological messages to target cells. *PLoS ONE*.

[36] Wang X, Gerdes H-H (2012). Long-distance electrical coupling via tunneling nanotubes. *Biochimica et Biophysica Acta: Biomembranes*.

